# Preparation of CaCO_3_-TiO_2_ Composite Particles and Their Pigment Properties

**DOI:** 10.3390/ma11071131

**Published:** 2018-07-03

**Authors:** Sijia Sun, Hao Ding, Xifeng Hou

**Affiliations:** Beijing Key Laboratory of Materials Utilization of Nonmetallic Minerals and Solid Wastes, National Laboratory of Mineral Materials, School of Materials Science and Technology, China University of Geosciences (Beijing), Xueyuan Road, Haidian District, Beijing 100083, China; ssjcugb@163.com (S.S.); houxifeng3204114@126.com (X.H.)

**Keywords:** CaCO_3_-TiO_2_, composite particle, pigment, wet grinding

## Abstract

CaCO_3_-TiO_2_ composite particles were prepared with calcium carbonate (CaCO_3_) and TiO_2_ in stirred mill according the wet grinding method. The pigment properties, morphology, and structure of CaCO_3_-TiO_2_ composite particles and the interaction behaviors between CaCO_3_ and TiO_2_ particles were explored. In the CaCO_3_-TiO_2_ composite particles, TiO_2_ is uniformly coated on the surface of CaCO_3_ and the firm combination between CaCO_3_ and TiO_2_ particles is induced by the dehydration reaction of surface hydroxyl groups. CaCO_3_-TiO_2_ composite particles have similar pigment properties to pure TiO_2_. The hiding power, oil absorption, whiteness and ultraviolet light absorption of composite particles are close to those of pure TiO_2_. The application performance of CaCO_3_-TiO_2_ composite particles in the paint is consonant with their pigment properties. The contrast ratio of the exterior paint containing CaCO_3_-TiO_2_ composite particles is equivalent to that of the paint containing the same proportion of pure TiO_2_.

## 1. Introduction

CaCO_3_ is an important inorganic mineral material. Especially, the CaCO_3_ powder material prepared with various non-metallic minerals, such as aragonite, calcite, and some rocks with calcite as the main ingredient component, has many advantages, such as high purity, high whiteness, good compatibility with organic and inorganic matrices, and low cost [1,2]. Therefore, CaCO_3_ has become the most widely used filler in many industrial products such as plastic, paint, and paper due to its obvious cost advantage compared to most non-metallic minerals [3,4]. In order to further increase the utilization value of CaCO_3_ and achieve the efficient utilization of mineral resources, it is necessary to develop CaCO_3_-based composite with oxides, such as TiO_2_ [5,6]. Recently, the composite pigment prepared by coating TiO_2_ particles on the surface of CaCO_3_ particles, had received wide attention [7]. The composite pigment can not only increase the utilization efficiency of pigment TiO_2_, but also reduce the consumption of pigment TiO_2_. The preparation of the composite pigment will enhance the comprehensive utilization of titanium resource [8,9,10].

TiO_2_-coated mineral materials are generally prepared by hydrolysis precipitation coating method [11,12]. Zhou [13] prepared the TiO_2_-coated barite composite pigments through the hydrolysis of TiOSO_4_ on the barite surface. Ninness [14] and Lu [15] prepared TiO_2_-coated kaolin composite pigments and Gao prepared the anatase and rutile TiO_2_-coated mica composite pigments through the hydrolysis coating of TiCl_4_ [16,17]. However, a strong acid is produced during the hydrolysis process of titanium salts. CaCO_3_ is instable in acid media due to its poor acid fastness. Consequently, the CaCO_3_-based composite could not be prepared through the hydrolysis of titanium salts such as TiOSO_4_. Hence, several methods were developed to prepare CaCO_3_-TiO_2_ composites. Tanabe [18] prepared CaCO_3_-TiO_2_ composite particles through carbonation in the TiO_2_ system. Liu [19] and Sun [20] prepared CaCO_3_-TiO_2_ composite particles by the sol-gel method and hydrophobic agglomeration method, respectively. However, the above mentioned methods are complicated and expensive, and can hardly realize large-scaled industrial production of CaCO_3_-TiO_2_ composite particles.

The mechanochemical method is widely used to prepare composite materials due to its simple process, low cost, and low pollution. Through the ultra-fine grinding of mineral particles, the surface of mineral particles can be activated, thus slightly changing the crystal structure and physicochemical properties of the surface. Consequently, the interfacial reaction between particles is strengthened to induce the combination. Wang [21] and Chen [22] respectively prepared barite/TiO_2_ and TiO_2_-coated wollastonite composite pigments by the mechanochemical method, and both composite pigments exhibited similar pigment properties to TiO_2_. However, the combination between barite and TiO_2_ was induced by electrostatic interactions. Therefore, the combination effect was not firm enough and needs to be enhanced. The light adsorption property or the application performance of the composite pigments was not explored. In this study, CaCO_3_-TiO_2_ composite particles were prepared in the water system according to the mechanochemical method. In addition, the pigment properties, light absorption properties, morphology, and structure of the prepared CaCO_3_-TiO_2_ composite particles were studied and the combination feature and mechanism between TiO_2_ and CaCO_3_ particles were also investigated. Additionally, the application performances of CaCO_3_-TiO_2_ composite particles in paint were evaluated.

## 2. Methods

### 2.1. Raw Materials

CaCO_3_ raw material used as the substrate in this study was produced in Huadian Quanxing Mining Co., Ltd. Manufacturer, Huadian, Jilin Province, China. The purity, hiding power, whiteness, and average particle size of the raw material are respectively 100% (indicated by X-ray diffractometer), 185 g/m^2^, 97.82%, 1.21 μm (d_50_), and 4.87 μm (d_90_), indicating that the CaCO_3_ raw material has the poor hiding property and high whiteness. The TiO_2_ raw material used in the experiment is commercially available rutile TiO_2_ produced by sulfuric acid method (Billionschem Co., Ltd., Jiaozuo, China). The whiteness, hiding powder, oil absorption and average particle size of TiO_2_ are respectively 96.9%, 14.61 g/m^2^, 26.06 g/100 g, 0.37 μm (d_50_) and 0.55 μm (d_90_), indicating the excellent pigment properties of TiO_2_ raw material. Chemically pure linseed oil (Zhengzhou Tianma Art Paints Co., Ltd., Zhengzhou, China) and distilled water were also used (Beijing Jinxin Hengda Technology and Trade Co., Ltd., Beijing, China).

### 2.2. Preparation

#### 2.2.1. Preparation of the CaCO_3_-TiO_2_ Composite Particles

CaCO_3_-TiO_2_ composite particles were prepared with CaCO_3_ and TiO_2_ in stirred mill by wet grinding. Firstly, the CaCO_3_ slurry and TiO_2_ slurry were prepared. CaCO_3_ and TiO_2_ were respectively mixed with water and sodium polyacrylate (dispersant) according to a certain proportion and stirred by a high-speed dispersion machine. Secondly, the CaCO_3_ and TiO_2_ slurries were mixed together and ground by a GSDM-S3 type ultrafine grinding mill (3 L, Beijing gosdel power&technology Co. Ltd., Beijing, China) with a speed of 1200 r/min and the weight ratio of ball to powder was 4:1. After stirring at room temperature and pH = 8 for 90 min, the composite slurry was obtained. Finally, the CaCO_3_-TiO_2_ composite particles were obtained after the CaCO_3_-TiO_2_ composite slurry was dried at 100 °C for 12 h (Electric thermostaticdrying oven, Tianjin Taisite Instrument Co., LTD, Tianjin, China).

#### 2.2.2. Preparation of the Construction Paint for Exterior Wall

The construction paints for exterior wall was prepared respectively with CaCO_3_-TiO_2_ composite particles and TiO_2_ particles as pigments. The raw materials were added into a high-speed mixer sequentially according to a certain proportion and then stirred to form a stable paint. In the paint, the TiO_2_ or the CaCO_3_-TiO_2_ composite particles were used as white pigment, they imparts opacity to the coating by bonding with the film-forming materials. The ground calcium carbonate was used as the main filler, playing a role in filling and reducing the cost of coatings. The total content of white pigment and ground calcium carbonate was 35.5 wt %. Besides, the acrylic emulsion was used as a film former, which allows the coating to be firmly adhered to the substrate to form a continuous film. Several additives including wetting agent, water, dispersant, pH regulator, defoamer, film-forming additive, leveling agent, and thickener were added to adjust the coating performance.

### 2.3. Characterization

#### 2.3.1. Structure and Property Characterization

We observed the morphology of CaCO_3_, TiO_2_, and CaCO_3_-TiO_2_ composite particles by scanning electron microscope (SEM, S-3500N, HITACHI, Tokyo, Japan) under an energy dispersive spectroscope at 5.0 kV and transmission electron microscope (TEM, FEI Tecnai G2 F20, Portland, OR, USA) under an acceleration voltage of 300 kV. For morphological observation, the powder samples were dispersed in ethanol solvent and sonicated for 10 min, and then the suspensions were directly dropped to the conductive adhesive or copper net for natural drying. The phase analysis were carried out on the X-ray diffractometer (XRD, D8 ADVANCE, BrukerAXS GmbH, Karlsruhe, Germany) with Cu Kα radiation (λ = 1.5406 Å) generated at 40 kV and 15 mA and a scanning rate of 8 °/min. The Fourier-transform infrared (FT-IR) spectra were recorded on an infrared spectroscope (Spectrum 100, PerkinElmer Instruments (Shanghai) Co., Ltd., Shanghai, China) in a scanning range of 4000–400 cm^−1^. The particle size was tested with centrifugal sedimentation (BT-1500, Dandong Bettersize Instrument (Liaoning) Co., Ltd., Liaoning, China). The whiteness was tested with a whiteness meter (SBDY-1, Shanghai Yuet Feng Instrument Co., Ltd., Shanghai, China). The ultraviolet (UV)-vis spectra of the prepared composite materials were obtained in the range of 200~800 nm on a TU-1901 double beam spectrophotometer (Beijing Presee General Instrument Co., Ltd. Beijing, China).

#### 2.3.2. Property Tests of the CaCO_3_-TiO_2_ Composite Particles and the as-Prepared Paint

The pigment properties of the CaCO_3_-TiO_2_ composite particles were evaluated in terms of oil absorption, hiding power, and relative hiding power and the previous testing methods [23,24] were adopted.

Oil absorption is an important index of pigments. According to the China National Standard GB/T 5211.15-2014 [25], the index refers to the minimum amount of varnish (linseed oil) required for completely wetting 100 g pigment.

Hiding power is another important index of pigments. It refers to the minimum amount of pigment required for completely covering a black and white checkerboard. The hiding power of a pigment can be tested according to the China National Industrial Standard HG/T 3851-2006 [26] (the Test Method of Pigment Hiding Power).

The relative hiding power (R, %) is defined as the ratio of the hiding power of the composite particles to that of pure TiO_2_ pigment. The R value can be calculated as:

R = (H_T_/H_CT_) × 100%, (1)

where H_T_ (g/m^2^) and H_CT_ (g/m^2^) are respectively the hiding power values of TiO_2_ and the CaCO_3_-TiO_2_ composite particles.

The value of ΔR calculated by Equation (2) represents the increase in the hiding power of TiO_2_ caused by the combination with CaCO_3_:

ΔR = R − R_0_ (2)

where R_0_ is the mass ratio of TiO_2_ to the composite particles.

According to the China National Standard GB/T 23981-2009 [27], the contrast ratio of the construction paint for exterior wall (C) can be tested according to the following method. A coating film with a certain thickness was firstly coated on the standard black-and-white plate and then the reflectivity values of black (R_B_, %) and white regions (R_W_, %) were tested with a reflectivity measuring instrument (C84-III, Tianjing Jinke Material Testing machine Co., Ltd., Tianjing, China). Finally, the contrast ratio of coating film can be obtained by Equation (3) [23]:

C = R_B_/R_W_ (3)

The total color difference (ΔE) between the paint containing CaCO_3_-TiO_2_ composite particles and the paint containing TiO_2_ pigment can be calculated by Equation (4):

ΔE = [(L_T_* − L_CT_*)^2^ + (a_T_* − a_CT_*)^2^ + (b_T_* − b_CT_*)^2^]^1/2^ (4)

where the L_T_*, a_T_*, and b_T_* represent the chromaticity values of coating films containing TiO_2_ particles; L_CT_*, a_CT_*, and b_CT_* represent the chromaticity value of coating films containing CaCO_3_-TiO_2_ composite particles. All the chromaticity values were measured with a portable integrating sphere spectrophotometer (X-Rite Sp60, X-Rite (Shanghai) International Trade Co., Ltd., Shanghai, China).

## 3. Results and Discussion

### 3.1. Morphology of CaCO_3_-TiO_2_ Composite Particles

**Figure 1:**
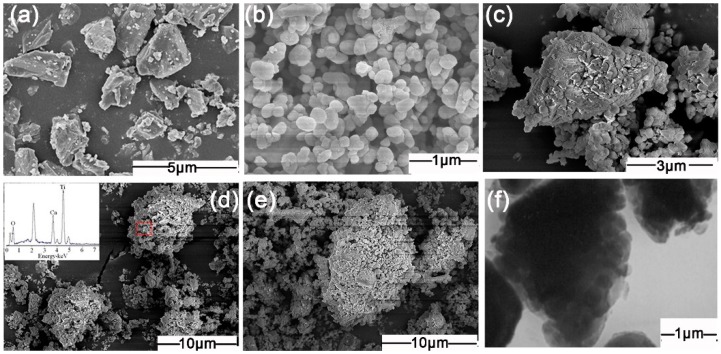
Scanning electron microscopy (SEM) and transmission EM (TEM) images of CaCO_3_, TiO_2_, and CaCO_3_-TiO_2_. (**a**) SEM image of CaCO_3_; (**b**) SEM of TiO_2_; (**c**–**e**) SEM image of CaCO_3_-TiO_2_ with TiO_2_ mass ratio of 30%, 40%, and 50%; (**f**) TEM of CaCO_3_-TiO_2_ (50%).

The uniformity and completeness of TiO_2_ coating on the surfaces of minerals are important influencing factors of the pigment properties of minerals-TiO_2_ composite particles. Therefore, we investigated the morphology of CaCO_3_ and TiO_2_ raw materials, as well as CaCO_3_-TiO_2_ composite particles with different mass ratios of TiO_2_. Corresponding SEM and TEM images are shown in Figure 1.

In Figure 1a, the bulk CaCO_3_ particles with the size of 1–3 μm were obvious and the surfaces of CaCO_3_ particles were smooth, without covering. In Figure 1b, the granular TiO_2_ particles with the particle size of 0.2–0.3 μm showed good dispersivity. However, after CaCO_3_ and TiO_2_ particles were ground together (Figure 1c–e), many fine particles were uniformly coated on the surfaces of CaCO_3_, and the smooth surface of CaCO_3_ became rough. As indicated by the Energy Dispersive Spectrometer (EDS) results (Figure 1d), the particles coated on the surfaces of CaCO_3_ should be TiO_2_. Apparently, the CaCO_3_-TiO_2_ composite particles were characterized by the uniform TiO_2_ coating on the surface of CaCO_3_. Consequently, the CaCO_3_-TiO_2_ composite particles should possess the properties of TiO_2_, such as the similar pigment properties to that of TiO_2_.

The uniformity and completeness of the TiO_2_ coating on the surfaces of CaCO_3_ particles increased accordingly when the mass ratio of TiO_2_ increased from 30% to 50% (Figure 1c–e). Particularly, when the mass ratio of TiO_2_ increased to 50%, the surfaces of the CaCO_3_ particles were almost completely covered by TiO_2_ particles. Additionally, as shown in the TEM image (Figure 1f), the CaCO_3_-TiO_2_ composite particles are composed of the particles characterized by uniform and dense TiO_2_ coating on the surface of CaCO_3_.

### 3.2. Binding Properties of CaCO_3_ and TiO_2_ Particles

#### 3.2.1. XRD Analysis

**Figure 2:**
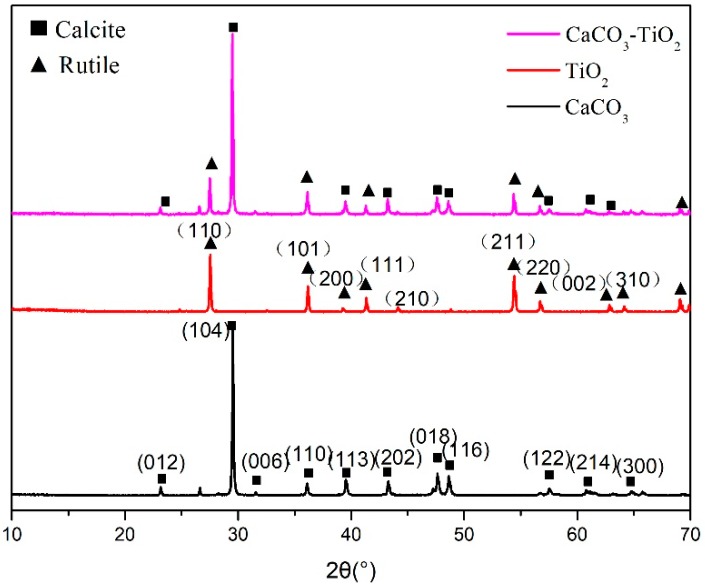
X-ray diffractometer (XRD) patterns of CaCO_3_, TiO_2_, and CaCO_3_-TiO_2_ composite particles.

Figure 2 shows the XRD pattern of CaCO_3_, TiO_2_, and CaCO_3_-TiO_2_ composite particles with the TiO_2_ mass ratio of 50%. There were only calcite and rutile diffraction peaks in the XRD pattern of CaCO_3_-TiO_2_ composite particles, indicating that the composite particles were still composed of caclite and rutile TiO_2_, and no new phase was produced in the preparation process of the composite particles. Meanwhile, the complete crystal phases of the CaCO_3_ and TiO_2_ materials remained without any changes. Therefore, it can be inferred that the binding of CaCO_3_ and TiO_2_ particles should occur at the interfacial region of the particles, whether the binding is of a chemical or physical nature.

#### 3.2.2. Infrared Spectral Analysis

**Figure 3:**
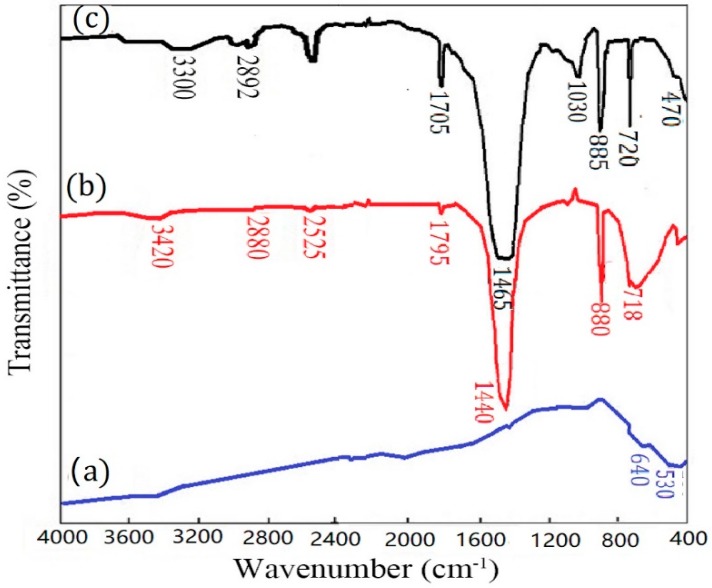
Fourier-transform infrared spectra (FT-IR) spectra of (**a**) CaCO_3_, (**b**) TiO_2_, and (**c**) CaCO_3_-TiO_2_ composite particles.

In addition to the coating behaviors of TiO_2_, including completeness and orderliness, on the surfaces of mineral particles, the binding properties and binding strength between mineral particles and TiO_2_ particles are also important influencing factors of the properties of the TiO_2_-coated composite particle pigments. In order to investigate the binding properties between TiO_2_ and CaCO_3_ particles, the infra-red (IR) spectra of TiO_2_, CaCO_3_, and CaCO_3_-TiO_2_ composite particles were tested and analyzed (Figure 3). Figure 3a displays several peaks in the range of 400~700 cm^−1^, which are ascribed to the stretching vibrations of Ti-O-Ti bonds. These peaks were characteristic peaks of TiO_2_ [28,29]. In Figure 3b, the absorption peaks at 880 cm^−1^ and 718 cm^−1^ were ascribed to the characteristic absorption peaks of CO_3_^2−^ in CaCO_3_ [30,31]. Meanwhile, the peak at 3420 cm^−1^ was ascribed to the hydroxyl groups formed by the reaction between H_2_O and the ions on CaCO_3_ surface such as Ca^2+^ and CO_3_^2−^. Several changes were observed in the IR spectrum of CaCO_3_-TiO_2_ composite particles (Figure 3c), compared to that of the raw materials. Firstly, the absorption peak of CO_3_^2−^ in CaCO_3_ appeared at 1465 cm^−1^, showing a shift and broadening phenomenon compared with the absorption peak at 1440 cm^−1^ in the infrared spectrum of CaCO_3_. The shifted absorption peak indicated that the chemical environment of CO_3_^2−^ changed due to the reaction with other materials, and the broadened absorption peak showed that the association degree between CaCO_3_ particles increased. Secondly, compared to the characteristic peak of hydroxyl groups at 3420 cm^−1^ in CaCO_3_ raw materials (Figure 3b), the characteristic peak of water and hydroxyl groups at 2892~3300 cm^−1^ in Figure 3c [32] was broadened and shifted in the direction of the low wave number. Thirdly, there is a clear absorption peak at 1030 cm^−1^ in Figure 3c, indicating that there might be a chemical bonding behavior related to the formation of Ti-O-Ca bond. According to the above analysis, it can be inferred that the combination of CaCO_3_ and TiO_2_ should be realized by the chemical interaction between hydroxyl groups on particle surfaces, so this combination should be firm. Undoubtedly, based on the uniform coating of TiO_2_ on the surface of CaCO_3_, the strong combination strength between CaCO_3_ and TiO_2_ largely determines the good pigment properties of CaCO_3_-TiO_2_ composite particles.

#### 3.2.3. Mechanism Analysis

The hydration behavior of unsaturated ions on the cleavage planes of CaCO_3_ and TiO_2_ can explain the formation of surface hydroxyl groups. The calcite CaCO_3_, which belongs to the tripartite crystal system, is completely cleaved on the plane (10$\bar{1}$1), and the unsaturated Ca^2+^ and CO_3_^2−^ ions are exposed. The unsaturated Ca^2+^ on CaCO_3_ surface will undergo the following hydration reactions:

Ca^2+^ + H_2_O → Ca(OH)^+^ + H^+^ (5)

Ca(OH)^+^ + H_2_O → Ca(OH)_2_ + H^+^ (6)

The above hydration reactions of Ca^2+^ are related to the pH value. The larger the pH value is, the stronger the hydration effect is [33,34]. Therefore, the hydration reaction of CaCO_3_ is intense. Obviously, there should be a certain amount of hydroxyl groups on the surfaces of CaCO_3_ generated in the hydration reactions of Ca^2+^ and CO_3_^2−^. As for TiO_2_, the hydration reactions of Ti^4+^ are intense. Hydrolysis reactions and corresponding constants [35] are provided as follows:

Ti^4+^ + H_2_O ⇆ Ti(OH)^3+^ + H^+^, pk_1_ = 14.15 (7)

Ti(OH)^3+^ + H_2_O ⇆ Ti(OH)_2_^2+^ +H^+^, pk_2_ = 13.73 (8)

Ti(OH)_2_^2+^ + H_2_O ⇆ Ti(OH)_3_^+^ + H^+^, pk_3_ = 13.39 (9)

Ti(OH)_3_^+^ + H_2_O ⇆ Ti(OH)_4_ + H^+^, pk_4_ = 13.06 (10)

Therefore, a large amount of hydroxyl groups are formed on the TiO_2_ surface due to the intense hydrolysis of Ti^4+^. Consequently, it can be inferred that CaCO_3_ and TiO_2_ are combined together by the reaction of hydroxyl groups on their surfaces.

**Figure 4:**
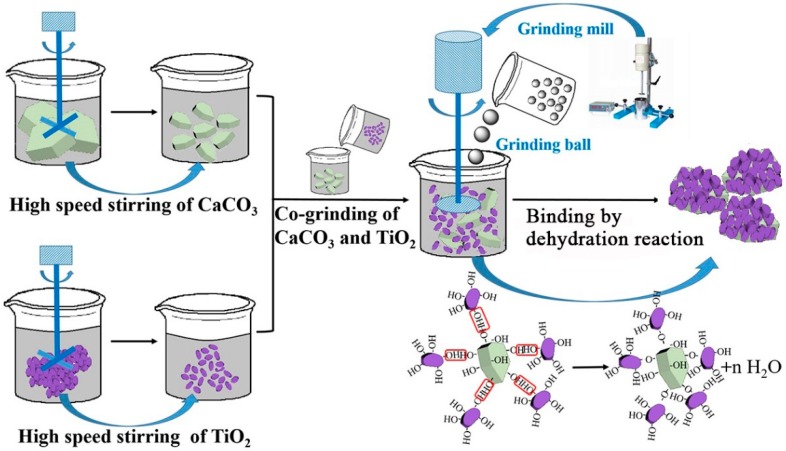
Preparation mechanism of CaCO_3_-TiO_2_ composite particles.

Based on the above analysis, the preparation mechanism of CaCO_3_-TiO_2_ composite particles by the mechanochemical method can be summarized as follows (Figure 4). First, through the high speed stirring of raw materials, the CaCO_3_ and TiO_2_ particles can be further dispersed, and then the hydration, dehydration, and other reactions between particles in aqueous media can be promoted because the slight distortion of the lattice of mineral particles and the activation of the particle surface during the grinding process enhances the binding between the surfaces [36]. Second, the high energy imported in the co-grinding process can increase the chance of the collision between CaCO_3_ and TiO_2_ particles, overcome the energy barrier of the repulsive interaction between the two particles, and reduce the distance between particles below the range allowing the interaction between functional groups on particle surface. Finally, the firm combination of CaCO_3_ and TiO_2_ particles can be achieved by the dehydration reaction among the hydroxyl groups on particle surface.

### 3.3. Properties of CaCO_3_-TiO_2_ Composite Particles

#### 3.3.1. Pigment Properties

**Figure 5:**
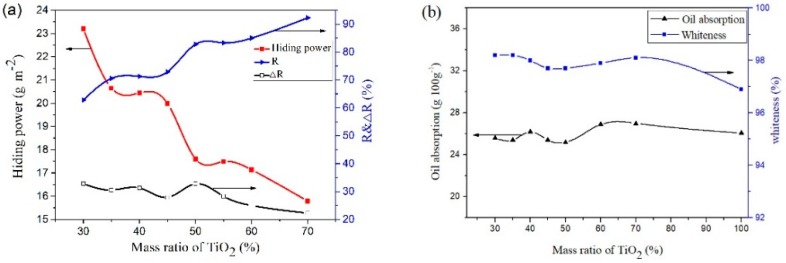
Pigment properties of CaCO_3_-TiO_2_ composite particles with different mass ratios of TiO_2_. (**a**) Hiding power (R) and relative hiding power (ΔR), (**b**) Oil absorption and whiteness.

Figure 5 shows the pigment properties of CaCO_3_, TiO_2_ raw materials, and the CaCO_3_-TiO_2_ composite materials with different TiO_2_ mass ratios, including hiding power, relative hiding power (R), the increased ratio of hiding power (ΔR), oil absorption, and whiteness.

As shown in Figure 5a, CaCO_3_-TiO_2_ composite particles exhibited much better hiding properties than CaCO_3_. When the mass ratio of TiO_2_ in the CaCO_3_-TiO_2_ composite was only 30%, its hiding power reached about 23 g/m^2^, which shows much stronger hiding properties than the CaCO_3_ raw material (185 g/m^2^). When the mass ratio of TiO_2_ increases to 50%, the hiding power and relative hiding power of CaCO_3_-TiO_2_ composite are 17.59 g/m^2^ and 82.80% (R), which was 32.80% (ΔR) higher than that of TiO_2_ (R_0_). As expected, the hiding power and relative hiding power of the CaCO_3_-TiO_2_ composite respectively reached 15.79 g/m^2^ and 92.27% when the TiO_2_ mass ratio increased to 70%, indicating that the CaCO_3_-TiO_2_ composite have obtained the excellent pigment properties equivalent to that of TiO_2_ pigment. Obviously, the coating of TiO_2_ on the surface CaCO_3_ weakened the properties of CaCO_3_ and induced the composite particles to exhibit the properties of TiO_2_. The abovementioned results indicated that the TiO_2_ particles in the CaCO_3_-TiO_2_ composite exhibited the higher utilization efficiency compared to the pure rutile TiO_2_ pigment because the coating structure improved the dispersion of titanium dioxide and the synergistic effect of calcium carbonate as a carrier. Meanwhile, the whiteness of CaCO_3_-TiO_2_ composite particles was also close to that of CaCO_3_ and TiO_2_, and the oil absorption of CaCO_3_-TiO_2_ composite was similar or less than that of TiO_2_.

#### 3.3.2. Optical Property of CaCO_3_-TiO_2_ Composite Particles

**Figure 6:**
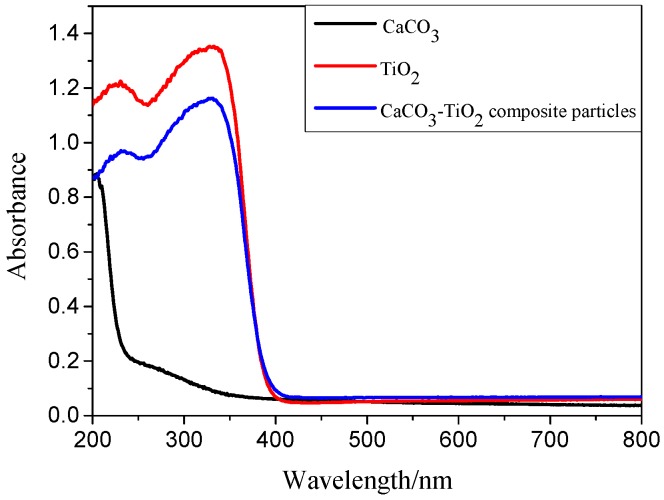
UV-vis absorption spectra of CaCO_3_, TiO_2_, and CaCO_3_-TiO_2_ composite particles.

The UV-vis absorption spectra of CaCO_3_-TiO_2_ composite particles, CaCO_3_ and TiO_2_ raw materials are shown in Figure 6. All samples almost showed no absorption of visible light in the wavelength range of 400–800 nm, reflecting their characteristics as white inorganic material. However, the three samples are different in the absorption of ultraviolet light in the wavelength range of 200–400 nm. CaCO_3_ raw materials exhibited almost no light absorption, but TiO_2_ and CaCO_3_-TiO_2_ composite particles exhibited strong light absorption. CaCO_3_-TiO_2_ composite particles exhibited less light absorption in the wavelength range of 200–350 nm than TiO_2_. Unlike CaCO_3_, the CaCO_3_-TiO_2_ composite particles obtained the same light absorption property as that of TiO_2_ and the result was in agreement with the previous report [5]. The abovementioned results not only indicated that the CaCO_3_-TiO_2_ composite particles obtained excellent UV resistance stability, but also reflected the mechanism that TiO_2_ particles coated on the surface of CaCO_3_ particles endowed the composite particles with the similar properties of TiO_2_.

#### 3.3.3. Properties of the Exterior Paint Containing CaCO_3_-TiO_2_ Composite Particles

**Figure 7:**
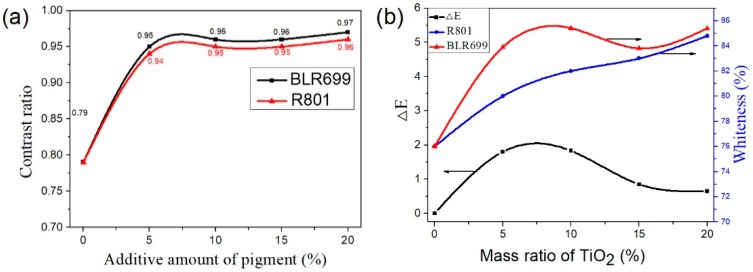
Properties of the two exterior paints respectively containing R-801 and BLR-699. (**a**) Contrast ratio of paint; (**b**) Whiteness and chromatic aberration of paint.

**Figure 8:**
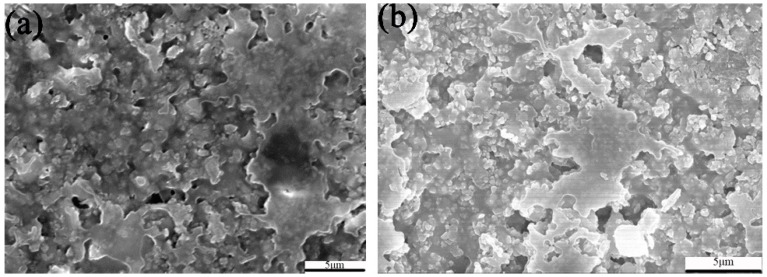
SEM images of two exterior paints respectively containing R-801 (**a**) and BLR-699 (**b**).

Figure 7 shows the property of the exterior paint containing CaCO_3_-TiO_2_ composite particles (R-801, the mass ratio of TiO_2_ is 70%) and rutile TiO_2_ (BLR-699). As shown in Figure 7a, when the addition proportions of R-801 and BLR-699 in the paint were only 5%, the contrast ratios of the obtained paints respectively reached 0.94 and 0.95, and the two values were close. Moreover, the abovementioned contrast ratios of the paint were higher than the contrast ratio (0.93) of superior products required in China National Standard of Exterior Paints (GB/T 9756-2009) [37]), indicating that R-801 and BLR-699 exhibited excellent pigment properties when they were applied in the paint. When the addition proportions of R-801 and BLR-699 in the paint increased to 10% or more, the contrast ratio of the paint was increased slightly. The contrast ratio of the paint containing R-801 was always equivalent to that of the paint containing BLR-699, indicating that the application of CaCO_3_-TiO_2_ composite particles as the pigment in the paint for exterior wall could achieve the equivalent covering effect to the paint containing the same proportion of TiO_2_. When the addition proportion of pigments in the paint is 5% or 10%, the whiteness of the paint containing R-801 was always lower than that of the paint containing the same proportion of BLR-699 (Figure 7b). However, when the addition proportion of pigments increased to 15% and 20%, the whiteness of the paint containing R-801 was close to that of the paint containing BLR-699 and the color difference value (ΔE) was reduced to about 0.7. As the addition proportion of pigment in the paint for exterior wall is generally high, the comprehensive performance of the paint containing R-801 is equivalent to that of the paint containing BLR-699. As shown in the SEM images of the dry paints respectively containing R-801 and BLR-699 (Figure 8), the two samples all present a continuous form composed of solid particles, such as the polymer formed after emulsion evaporation, binder, pigments, and fillers, indicating that both the R-801and BLR-699 have good compatibility with emulsion.

## 4. Conclusions

CaCO_3_-TiO_2_ composite particles were prepared with CaCO_3_ and TiO_2_ in stirred mill according to the wet grinding method. The composite particles are characterized by the uniform TiO_2_ coating on the surface of CaCO_3_. The CaCO_3_ and TiO_2_ particles were firmly combined together through the dehydration reaction of hydroxyl groups on their surfaces.

CaCO_3_-TiO_2_ composite particles exhibited similar pigment properties and light absorption property to TiO_2_. Moreover, the CaCO_3_-TiO_2_ composite particles also showed excellent application performance. The contrast ratio of the exterior construction paint containing CaCO_3_-TiO_2_ composite particles as the pigment was similar to that of paint containing the same proportion of TiO_2_, and was higher than the contrast ratio (0.93) of superior products required in the China National Standard of Exterior Paints (GB/T 9756-2009 [36]).
